# Hunting for Genes Underlying Emotionality in the Laboratory Rat: Maps, Tools and Traps

**DOI:** 10.2174/1570159X20666220901154034

**Published:** 2023-07-10

**Authors:** André Ramos, Natalli Granzotto, Rafael Kremer, Ariela Maína Boeder, Julia Fernandez Puñal de Araújo, Aline Guimarães Pereira, Geison Souza Izídio

**Affiliations:** 1 Behavior Genetics Laboratory, Department of Cell Biology, Embryology and Genetics, Center of Biological Sciences, Federal University of Santa Catarina, Florianopolis, Brazil;; 2 Graduate Program of Pharmacology, Center of Biological Sciences, Federal University of Santa Catarina, Florianopolis, Brazil;; 3 Graduate Program of Developmental and Cellular Biology, Center of Biological Sciences, Federal University of Santa Catarina, Florianopolis, Brazil

**Keywords:** Behavior genetics, anxiety, hyperactivity, linkage analysis, quantitative trait loci, quantitative trait genes, rats

## Abstract

Scientists have systematically investigated the hereditary bases of behaviors since the 19th century, moved by either evolutionary questions or clinically-motivated purposes. The pioneer studies on the genetic selection of laboratory animals had already indicated, one hundred years ago, the immense complexity of analyzing behaviors that were influenced by a large number of small-effect genes and an incalculable amount of environmental factors. Merging Mendelian, quantitative and molecular approaches in the 1990s made it possible to map specific rodent behaviors to known chromosome regions. From that point on, Quantitative Trait Locus (QTL) analyses coupled with behavioral and molecular techniques, which involved *in vivo* isolation of relevant blocks of genes, opened new avenues for gene mapping and characterization. This review examines the QTL strategy applied to the behavioral study of emotionality, with a focus on the laboratory rat. We discuss the challenges, advances and limitations of the search for Quantitative Trait Genes (QTG) playing a role in regulating emotionality. For the past 25 years, we have marched the long journey from emotionality-related behaviors to genes. In this context, our experiences are used to illustrate why and how one should move forward in the molecular understanding of complex psychiatric illnesses. The promise of exploring genetic links between immunological and emotional responses are also discussed. New strategies based on humans, rodents and other animals (such as zebrafish) are also acknowledged, as they are likely to allow substantial progress to be made in the near future.

## 1 INTRODUCTION

Let us be honest: identifying genes influencing phenotypes that vary continuously within any given species is long, arduous and risky. If some of us thought otherwise, we were probably excessively excited with the possibility of merging, for the first time, Mendelian, quantitative and molecular genetic strategies back in the early 1990s. Perhaps we were not paying enough attention to our history lessons in such a promising conceptual and methodological context. We were (and we are), indeed, a privileged generation, for having, at last, the technological means of explaining biologically some of the fundamental phenomena that the founders of Genetics could only explain mathematically (and often with little consensus). Had Gregor Mendel chosen, in his famous hybridization experiments with peas [1], seven continuous variables instead of seven easily classifiable characteristics and maybe the science of heredity would have to wait for a few more decades before coming to light.

Studies on the inheritance of continuous variation had started long before the official dawn of Genetics. Darwin’s cousin, Francis Galton, produced much of the statistical and conceptual bases for Biometrics after the publication of Mendel’s seminal work, but before its discovery in 1900 [2]. In those years, but also in the first decades of the 20^th^ century, heated debates attempted to determine whether continuously varying traits were compatible or not with the evolutionary effects of natural selection [3]. Mendelian patterns of inheritance seemed to support the “mutationist” notion that quantitative characters, when influenced by selection, could not produce long-lasting and ever-progressing changes. For example, the Danish botanist Wilhelm Johannsen, one of the founders of Genetics and the creator of the terms “phenotype” and “genotype”, provided clear-cut evidence that both genetic and environmental factors underlay continuous biological variables, such as the weight of bean seeds. Nevertheless, he firmly believed that selection of such traits (which he did carry out) could not change phenotypes for more than a few generations and beyond the limits of the parental lines’ variation [3]. Such a hypothesis would imply that even highly complex traits, such as almost all behaviors, would be controlled by a relatively small number of genes. If that assumption was true, gene-hunting strategies, when applied to psychiatry, would have evolved faster than what we have seen in the past 30 years. As we easily recognize now, individual-gene approaches coupled with complex-trait analysis, in addition to being laborious and expensive, tend to fall short in terms of groundbreaking functional insights [4].

Interestingly, the first experimental evidence showing the highly elastic limits (and hence the highly polygenic nature) of quantitative-trait selection, has not come from experiments with peas or beans, but rather with the laboratory rat. Between 1907 and 1914, Willian Castle selected two contrasting lines for the width of coat-pigmented areas of the so-called “hooded” rats [3]. After 17 generations, animals from the “plus” and “minus” lines had reached such extreme contrasting phenotypes that they exceeded by far the pigmentation levels of the original parental population [5]. Such results had a tremendous impact on the understanding of genetics and evolution.

Even more relevant to the present review, however, the first selection experiment to be carried out by a psychologist, who was more interested in the physiological correlates of learning than in genetic selection itself, was also done with rats. The famous Tryon’s “maze-bright” and “maze-dull” rat lines were selected for 21 generations between 1926 and 1929 [3]. Thus, in a certain way, we are about to celebrate one century of behavior genetics using the rat as a model.

Tryon’s lines became so contrasting for the selected behavioral trait that there were hardly any doubts afterwards that, not only behaviors were partly inherited but also highly modifiable by selection, as well as highly polygenic. In the decades that followed, these seminal studies, in combination with abundant data from fruit flies, mice and humans, led to the consensus that: all behaviors were influenced by genetics; none was completely heritable; and most were affected by many genes with small effects [6]. Such discoveries have set the stage for the main challenge we would face much later, towards the end of the 20th century: how could we move beyond Mendelian and quantitative genetics and take advantage of the molecular revolution that took place in the preceding decades, to finally unravel the mysteries of neurobiological processes? From the abovementioned retrospective, one could suspect that there was still one piece missing in the intricate puzzle of approaches and techniques that could allow us to fill the gap between behavior and intra-cellular mechanisms. This missing part was “genetic mapping” combined with the use of molecular markers.

In 1910, Thomas Morgan located, for the first time, a specific gene in a particular chromosome of the fruit fly *Drosophila melanogaster*. Despite the skepticism of top genetic authorities such Johannsen and Bateson, who found the idea of chromosomes harboring genes as “inconceivable”, the world’s first genetic linkage map (described then as a “linear arrangement of linked factors”) was published by Morgan’s disciple, Alfred H. Sturtevant, in 1913 [7]. That concept, which remained essentially unchanged until the dawn of the genomic era, allowed the statistical association between phenotypic and molecularly-mapped genotypic variation.

For many years, rats had no complete linkage maps. With the discovery of hundreds of molecular markers known as microsatellites [8], the laboratory rat's first complete linkage map, comprising all chromosomes and 432 markers, was published in 1995 [9]. That was the main mapping tool available when we mapped the first loci related to emotionality in rats [10]. The average space between markers was 3.7 centiMorgans (cM) (or near 6.5 million base pairs, Mbp); only 103 known genes were included and many map positions were inaccurate. Nevertheless, only three years later, a new rat map containing more than 3, 000 markers was available [11]. Nowadays, over 44, 000 markers and 22, 000 protein-coding genes are mapped, according to the Rat Genome Database (RGD) [12]. This and many other more advanced technologies are available today. Yet, our crucial challenges still seem to reside in the genetic nature of quantitative traits, as understood one hundred years ago.

## 2 RATS AS GENETIC MODELS

The use of rats (*Rattus norvegicus*) in laboratory research started more than 170 years ago and grew exponentially during the 20^th^ century, which makes this species the earliest and most comprehensively studied animal in the history of biomedical sciences [13-15]. Among the many advantages of using rats as experimental models, we can mention their size, well-studied physiology, human-like diet, reproductive success rate, resistance to infections and, more recently, their completely sequenced genome [13, 16, 17]. Rats have a slightly smaller genome than humans (2.65 billion base pairs (bp) distributed over 21 pairs of chromosomes *vs*. 3.10 billion bp and 23 pairs of chromosomes, respectively) and mice (2.73 billion bp and 20 pairs of chromosomes) [12]. However, all three species have about the same number of genes, nearly 40% of which are very similar in structure and function [18].

Laboratory rats have been historically preferred by physiologists and, more recently, by a growing number of geneticists. The reproductive manipulation of rats allowed the development of both outbred (heterogeneous groups obtained by random crosses and presenting 99% of heterozygosity) and inbred (isogenic groups obtained by continuous inbreeding with 99% of homozygosity) lines or strains [13, 19]. Although outbred animals are more natural, developing isogenic strains was essential for the progress of genetic studies. Currently, there are more than 230 isogenic rat strains, some of which are phenotypically well characterized [20, 21]. Individuals within these strains have nearly identical genomes. Yet, when compared to other strains, they may display contrasting behavioral responses when submitted to emotionally challenging tests such as the open-field (OF) and the elevated plus-maze (EPM) [22]. Such characteristics, coupled with the intercrossing between strains, facilitate the search for correlations between phenotypic and genotypic variation throughout the genome.

Much of the phenotypic variation observable within all species, including humans and the laboratory rat, is quantitative and multifactorial. For example, human inter-individual differences in anxiety, mood, intelligence and drug sensitivity levels are influenced by many genes as well as by non-genetic factors. In other words, any given quantitative trait depends on the effect of multiple polymorphic genes interacting with a myriad of environmental factors [23].

There are two main phenotype-based strategies for performing genetic mapping and identifying genes for quantitative traits. One concerns Genome-Wide Association Studies (GWAS), commonly used in outbred populations and aim at associating Single Nucleotide Polymorphisms (SNP) with a given trait of interest. The other strategy involves genome-wide linkage analyses in mapping chromosomal regions influencing quantitative characteristics. This second method, called Quantitative Trait Loci (QTL) analysis, started near the end of the 1980s and aimed at elucidating the molecular mechanisms modulating clinically-relevant quantitative traits, most often using animal models [24-26].

## 3 QTL ANALYSIS

**Fig. (1):**
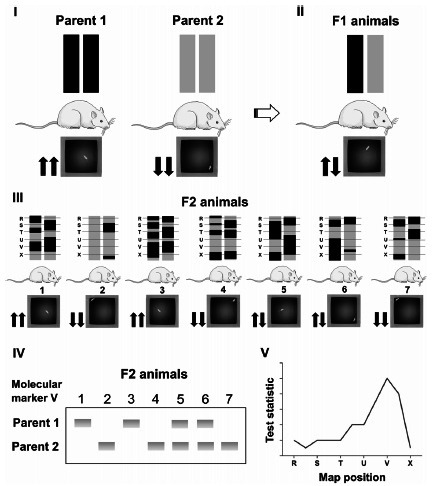
An example of the Quantitative Trait Loci (QTL) analysis method. (**I**) Two parental inbred rat strains contrasting for central locomotion in the open field are intercrossed to obtain heterozygous F1 animals (**II**). This generation is then intercrossed to produce segregating F2 animals (**III**). Rats from the F2 generation are then submitted to the open-field test (phenotyping) and then are genotyped with molecular markers distributed throughout the entire genome (**IV**). In this example, the hypothetical marker “V” is shown. For each molecular marker, F2 animals can be either homozygous or heterozygous. (**V**) The QTL analysis is finally performed to reveal the probability that any given genomic area influences the phenotype of interest, in this case, central locomotion in the open field. Also, in this example, the chromosome region bearing the molecular marker “V” has a high probability of being related to central locomotion in the open-field test, an emotionality-related behavior.

Typically, in QTL analysis, two strains displaying contrasting phenotypes must be intercrossed to produce an F1 generation, which is then used to generate F2 recombinant individuals (Fig. **1**). Strategies that are regularly used in QTL mapping may include backcrosses or intercrosses between isogenic, recombinant, congenic, or consomic strains [27, 28]. After the production of the F2 generation, the recombinant individuals are submitted to experimental procedures that evaluate the quantitative trait or traits being studied. Any aspect of the animal's biology can be assessed at this (phenotyping) stage. For example, it is possible to submit F2 animals to a battery of behavioral tests that assess experimental anxiety or emotionality, such as the OF, EPM, black and white box (BWB), or all of them combined [29]. Following phenotyping, it is necessary to extract and analyze the DNA from all F2 animals. The goal is to detect which parental line the genomic regions of each F2 animal come from (genotyping). Typically, this step is performed with hundreds of polymorphic molecular markers spread throughout the entire species’ genome [30]. Finally, with phenotypic and genotypic data available, the QTL analysis is carried out using specialized software, which reveals the likelihood of any genomic area influencing any measured continuous trait.

## 4 QTL STUDIES IN RATS

According to the RGD [12] more than 2, 300 QTLs are mapped in the rat genome. They include loci for behavioral measures of emotionality, motor activity, memory, drug abuse, preference and sensitivity, as well as numerous anatomical and physiological traits [10, 26, 30-42]. Verdugo and colleagues [43] performed a detailed review followed by a meta-analysis of several studies using a QTL/microarray approach on various rat strains. In this type of analysis, gene expression values obtained from microarrays are considered as quantitative traits. These traits will have their chromosomal locations mapped, being thus called expression QTL (eQTL).

Regarding psychiatric-relevant phenotypes, Moisan and Ramos have listed over 160 QTLs that had been mapped in rats until 2010, including their names, chromosome positions, strains and statistical significance [44]. By analyzing currently available data from the RGD, we have identified 167 QTLs for emotionality-related traits, 82 for stress responses, 56 for anxiety and 64 for locomotor activity, some of which overlap with each other [12].

In addition to the previously discussed classical inbred and outbred strains of rodents, recent initiatives have provided alternative genetic resources for QTL studies. In mice, for example, we highlight the panels of strains known as BXD (recombinant inbred animals from C57BL/6J and DBA/2J mice) and AXB/BXA (created from the crossing between AJ and C57BL/6J mice) [45-47]. In rats, a remarkable initiative was the Hybrid Rat Diversity Program (HRDP), which created a genetically and phenotypically diverse panel of more than 90 inbred strains to be used to map complex traits. It comprises the FXLE/LEXF rats (33 Japanese strains), the HXB/BXH rats (30 strains from the Czech Republic) and 35 fully sequenced and fully genotyped strains, providing a platform for reproducible experiments using both sexes [12, 48].

Commercial inbred strains such as Spontaneously Hypertensive Rats (SHR), Brown Norway (BN), Wistar Kyoto (WKY) and Fisher (F344) appear in several QTL studies involving stress, anxiety, depression and other emotional traits (Table **1**). As further discussed below, an F2 between Lewis (LEW) and SHR strains revealed a major QTL on chromosome 4, affecting locomotion in the center of the OF [49]. From this seminal discovery, a series of subsequent studies have aimed at isolating this genome region to further understand the psychopharmacological significance of its candidate genes.

## 5 THE DISCOVERY OF A MAJOR QTL ON RAT CHROMOSOME 4

In 1997, Ramos and colleagues [22] analyzed the behavior of six commercial isogenic strains of rats in classic apparatuses thought to measure experimental anxiety: the OF, EPM and BWB. In that seminal work, it was shown that the SHR and LEW strains were the most contrasting ones for a series of anxiety-related behaviors, without showing major differences in motor activity *per se* (differently from all other rodent models of anxiety that had been previously described) [49]. Therefore, these strains were proposed as a new genetic model for studying the neurobiological bases of anxiety.

LEW and SHR rats displayed major differences in their levels of locomotion in the center of the OF, where SHRs exhibited higher scores (which was suggestive of lower experimental anxiety), while LEW rats displayed lower exploration of this aversive area (thus suggesting higher experimental anxiety). Similar contrasting profiles were observed in the EPM and BWB, suggesting that the two strains differed consistently in anxiety tests. In a second study using F1 and F2 animals from an intercross between LEW and SHR rats, it was possible to determine that this phenotypic strain contrast was essentially conditioned by the animals’ genotypes [10, 22].

Subsequently, Ramos and colleagues [10] used the F2 generation of the intercross mentioned above to carry out a whole-genome QTL analysis. This work resulted in the mapping of the first QTLs for emotionality-related behaviors in rats. Two of them, originally named *Ofil1* and *Ofil2* (open field inner locomotion #1 and #2), influenced the approach/avoidance towards the OF central and aversive area. *Ofil1*, the stronger one, was later renamed *Anxrr16* (anxiety-related response #16) [12]. This exceptionally high-impact *locus* explained more than half of the total phenotypic variability of F2 animals. On the other hand, it was shown to cover almost half of chromosome 4, which contains more than one thousand protein-coding genes [49]. Interestingly, when the two alleles of this *locus* were inherited from the parental LEW strain (which displayed lower OF central locomotion when compared to SHR), F2 animals had higher levels of central exploratory behavior than rats inheriting both alleles from the parental SHR strain. This effect was opposite to what might be expected, but counterintuitive effects like this are relatively common in the context of QTL analyses [10]. Although the effects of *Anxrr16* were initially described only in females, later studies extended these effects to males as well [10, 65].

This region of rat chromosome 4 seems quite interesting from the point of view of preclinical behavior genetics. Research that followed those seminal studies demonstrated the importance of *Anxrr16* for a range of relevant behaviors. A study analyzing hybrid animals created from another cross between LEW and SHR rats showed that animals bearing two LEW copies of the Anxrr16 region (called high line) had higher exploratory levels in the OF than those carrying two SHR copies (called low line) [60]. As already mentioned, the effect of *Anxrr16* was extended to males, which, in that particular study, exhibited even greater phenotypic contrast than females. Furthermore, the aforementioned work corroborated the idea that this QTL was more related to emotional processing than locomotor behavior [60].

A further study using a very similar methodology demonstrated that *Anxrr16* could also affect ethanol consumption, although this effect was revealed only in females [62]. Other studies using descendants of LEW/SHR intercrosses extended the effects of *Anxrr16* to stress-induced analgesia and cocaine sensitization, both traits potentially related to emotionality [62, 66]. Izidio and colleagues [42] conducted an independent QTL analysis focused on rat chromosome 4 using F2 animals descending from different substrains of SHR and LEW rats. This study reported five additional QTLs for ethanol consumption, besides confirming the known anxiety-related effects and chromosome position of *Anxrr16*. Furthermore, the effect of this QTL was found in males as well, whereas, in females, an effect dependent on the estrous cycle could be observed.

## 6 THE SLA16 CONGENIC STRAIN

In order to better understand how the *Anxrr16* QTL influences emotionality-related behaviors, the production of a congenic strain was chosen as the main strategy in our laboratory. Animals of the LEW and SHR strains were intercrossed and the hybrid animals were continuously backcrossed with the SHR parental strain. Through a series of genotyping followed by a genetic selection of the breeders, after 10 generations of backcrossing (N10) the *Anxrr16* region was successfully transferred from the LEW strain into the SHR genetic background. From that point on, congenic animals started to be crossed among themselves and, after 20 generations (N10F20) of inbreeding, they became an isogenic strain that was named SHR.LEW-Anxrr16 (SLA16) [64]. This is, to our knowledge, the first congenic strain of rats specifically developed to investigate the genetic bases of emotionality-related behaviors.

Because SLA16 and SHR animals (which became the genetic controls of SLA16) are bred in the same animal facilities and under the same conditions, the behavioral differences between them can be attributed to the genes located within the differential genomic region (DGR), which comprises 81.9 Mbp. In the first report describing this new strain, De Medeiros and co-workers [64] confirmed the transgressive and counterintuitive effect of *Anxrr16*, revealing that SLA16 rats exhibited even less anxious-like behaviors than the parental SHR strain (which was already considered a model of low anxiety compared to other rat strains). In the study mentioned above, the SLA16 strain exhibited greater exploration of the aversive regions of various experimental apparatuses (OF, BWB, EPM and activity cage) when compared to their SHR controls. However, when tested in an activity cage after 22.5 h of habituation, differences between strains disappeared, suggesting that the effects of *Anxrr16* are related to a motor reaction to novelty rather than simple motor activity.

After the development of the SLA16 strain, a range of possibilities opened up to dissect the influence of *Anxrr16*, on emotional behaviors, and other clinically relevant phenotypes. A new QTL analysis conducted by Anselmi and colleagues [65], using F2 animals derived from SHR and LEW, demonstrated an overlap between *Anxrr16* and newly mapped *loci* for memory and learning. In that study, behaviors in a plus-maze discriminative avoidance task (PMDAT), object recognition (OR), spontaneous alternation (SA), and fear conditioning (FC) were co-analyzed with genotypic variation data. New QTLs for memory were reported in the same region as *Anxrr16*, but it was also possible to confirm the influence of these new QTLs in rats of the SLA16 strain. These data, once again, confirmed that SLA16 rats display higher exploration and lower anxious-like profile compared to SHR. It was also demonstrated, for the first time, that SLA16 rats have impaired cognitive performance when compared to SHR controls (which already perform poorly in memory tests, and are considered the gold standard genetic model of attention deficit hyperactivity disorder (ADHD)).

Pértile *et al*. [67] have evaluated the influence of *Anxrr16* on the responses to dopaminergic drugs in an OF test. In the first part of the work, an agonist (quinpirole) or an antagonist (haloperidol) of dopaminergic D2 receptors was administered intraperitoneally, while in its second part, the same drugs were administered into the ventral hippocampus. The study suggested, for the first time, that the influences of *Anxrr16* on OF behavior may be regulated by dopaminergic receptors, at least when they are manipulated systemically.

In another study, SHR and SLA16 rats were submitted to the triple test (an apparatus combining the OF, EPM and BWB) for five consecutive days, after repeated treatment with midazolam. It was shown that SLA16 females increased exploration of the open arms of the EPM over days, corroborating the notion that *Anxrr16* influences anxiety-like behaviors related to novelty [68].

Velazquez *et al*. [69] also evaluated SLA16 and SHR rats in a series of emotionality, memory and learning behavioral tests, as well as in biochemical essays to assess metabolic functions. Their report confirmed that *Anxrr16* exerts an influence on emotionality, memory and learning. Moreover, this study also demonstrated that SLA16 rats have higher levels of triglycerides (at 2 months of age) and cholesterol (at 2 and 8 months of age), as well as mild glucose intolerance when compared to SHRs.

The past twenty years of research on the influences of *Anxrr16* have confirmed it as a very promising genome region in the search for the molecular bases of emotional processes. Numerous behavioral reactions influenced by this *locus* are considered to be experimentally relevant to a variety of psychopathologies, not only related to anxiety and ADHD, but also to drug/ethanol abuse, memory and learning. Nevertheless, this promising QTL has not yet revealed its causal gene or genes up to this point.

## 7 FROM QTL TO QTG

Despite the progress mentioned above, narrowing down a very large genomic interval containing a QTL to a small interval harboring only one gene is neither simple nor guaranteed. First of all, as pointed out by Lander and Kruglyak [24], in order to be considered relevant, the results of any genetic mapping should be replicated by subsequent studies. Secondly, small sample sizes may fail to reveal a QTL or, on the contrary, may result in overestimating a QTL size effect [70]. Third, the QTL technique can only map differences that already exist between the parental lines used at the beginning of the process. In other words, highly relevant genome regions may not be revealed in any study involving a given pair of strains that do not differ for those important regions. Moreover, the phenotypes being studied are often influenced by a diversity of uncontrolled factors (*e.g*. sex, environment, or epistatic interactions), which can easily change over time. Finally, average QTLs range in length from 10 to 50 cM [71] and therefore, they can harbor thousands of genes and thousands of regulatory regions.

In order to overcome these difficulties and shorten the long path between identifying a QTL and discovering the corresponding Quantitative Trait Genes (QTG) (for example, through fastidious strategies of positional cloning and congenic strain production), one can take a shortcut and evaluate positional candidate genes. Good genes should map in the same chromosomal region of the QTL and their function should relate to the phenotypic trait under investigation [72]. Once identified, one can compare good candidate genes through DNA analysis (Southern Blot, SNP analysis, gene sequencing), messenger RNA (mRNA) expression (microarrays, Real-Time RT-PCR, Northern Blot, total gene expression analysis), quantification of the corresponding protein (Western Blot, immunohistochemistry) and bioinformatics.

However, even with this wide panel of techniques, identifying causal QTG remains highly challenging, especially for average QTL with relatively small phenotypic effects. In order to face this challenge, some authors propose to analyze the expression of genes mapped in the QTL region, from a biological tissue related to that trait [73]. Using this strategy, coupled with viral-mediated up and downregulation of the candidate gene, Gunduz-Cinar *et al*. [73] have identified a new gene influencing fear extinction in mice. Other authors suggest that a combination of gene expression analysis and statistical causal analysis can significantly reduce the number of relevant candidate genes [74]. Moreover, whole genome sequence data from the parental strains of selectively bred mice can be coupled with bioinformatics approaches to construct a tool for finely examining data from QTL experiments [75].

It is important to emphasize, however, that the development of consomic (when an entire chromosome is transferred from one strain to another) or congenic (such as the aforementioned SLA16 rats produced by our laboratory) strains provide experimental advantages that cannot be easily achieved with other strategies [20, 21, 76-78]. Using one of these approaches, Conti *et al*. [61] have developed a congenic strain for chromosome 8, from the crossing between SHR and BN rats. As a result, the authors were able to associate anxiety-related phenotypes to QTLs on chromosomes 2 and 3.

Ahmadiyeh *et al*. [55], using the Defensive Burying (DB) test, have found at least six significant QTLs for this stress-related behavior, some of which also influenced measures from other behavioral paradigms that reflected anxiety/emotionality. These and other studies (Table **1**) served as the basis for the development of the F344.WKY-Stresp10 (Fisher344.WistaKyoto-Stresp10) congenic strain, which has been used in the search for QTGs located near the Stresp10 QTL, on rat chromosome 6 [56].

Dumas *et al*. [50], using restraint-generated stress in recombinant strains, in parallel with gene expression analysis, found differential expression of heat shock protein (*hsp*) mRNA that mapped at known QTL on chromosomes 4 and 7, suggesting the *hstf1* gene (coding for a heat shock transcription factor) as a candidate for modulating the neurobiological reactions to stress. In Table **1**, we show additional work carried out with different strains and strategies using QTL and QTG approaches in rats.

## 8 SOME CANDIDATE GENES FOR *ANXRR16*

**Fig. (2):**
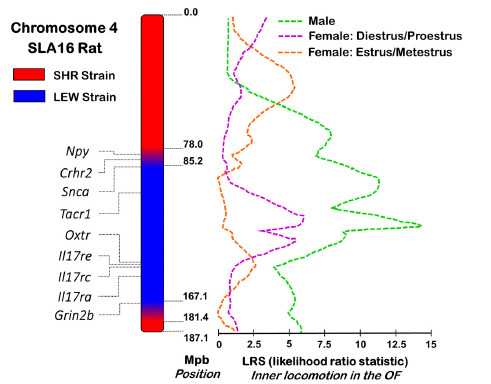
The *Anxrr16* QTL. This *locus* maps on rat chromosome 4 and has 81.9 Mbp, between the molecular markers D4Rat76 (85.2 Mbp) and D4Mgh11 (167.1 Mbp). The LRS (likelihood ratio statistic) values plotted herein are adapted from Izidio *et al*. [42]. LRS for F2 males and females (according to their estrous cycle) reflect the likelihood, which is calculated at every centiMorgan (cM), that each point of the genome influences central locomotion in the open field test.

The genome region containing *Anxrr16* has about 81.9 Mbp and it maps between the markers D4Rat76 and D4Mgh11, which are located on rat chromosome 4, at 85.2 and 167.1 Mbp, respectively [12]. In the congenic SLA16 strain, beyond the “pure-bred area” containing only LEW alleles, two flanking regions harbor alleles from both parental strains. These transition zones (which are always expected to exist) map between 78.0 and 85.2 Mbp and between 167.1 and 181.4 Mbp (Fig. **2**) [49, 64]. The creation of a congenic strain such as SLA16 allows the investigation of a target locus without the interference of the rest of the genome (differently from F2 populations). However, it is important to emphasize that the QTL strategy, unlike knockout and transgenic approaches, is based on natural genetic variations.

The DGR contains numerous genes of interest involved in emotional and stress responses, immunity, ethanol consumption and gabaergic, dopaminergic and glutamatergic pathways [79, 80]. Nine genes have caught our attention, either for their functional relevance or because of previous data involving LEW, SHR or SLA16 rats and their particular behavioral phenotypes (Table **2**). Yet, one must remember that QTLs do not harbor only genes, they also carry non-coding genomic variants that may play a key role in the observed QTL effect [81] Moreover, as one may observe in Fig. (**2**), the *Anxrr16* locus is composed of different QTL peaks. This suggests that different linked variants (*i.e*. genes or regulatory regions that are located in the same chromosome region) may be responsible for the overall locus effects [42].

### 8.1 Npy

NPY is a 36-amino-acid polypeptide that is phylogenetically conserved among species. It is one of the most abundant brain peptides and is expressed in multiple areas of the peripheral and central nervous system (CNS). It is known to interact with different subtypes of G-protein-coupled receptors, for example Y1, Y2, Y4 and Y5 [83-85]. NPY has an essential function in neurodevelopment, nutritional behavior, energy homeostasis, growth, circadian rhythm, vasoconstriction, angiogenesis, pain, learning and memory [86-92].

This polypeptide also plays an important role in anxiety and fear reduction, as well as in the relief and neuromodulation of stress adaptation processes in the hypothalamic-pituitary-adrenal (HPA) axis [84, 93]. In a study where ablation of NPY neurons in the nucleus accumbens (NAc) of mice was performed, researchers observed increased anxious-like behaviors in the OF and EPM tests [94]. In other studies [95-98], various rat strains have shown anxiolytic and sedative effects after intracerebroventricular administration of NPY. However, in the SHR strain, anxiolytic effects associated with increased locomotor activity have been observed [97].

The administration of Y1 receptor agonists in the amygdala of Wistar rats has reduced the amplitude of N-methyl-d-aspartate (NMDA) receptor-evoked excitatory postsynaptic currents as well as increased the amplitude of γ‐aminobutyric acid type A (GABAA) receptor-mediated inhibitory postsynaptic currents [99]. Additionally, concerning drug abuse, the activation of Y1 receptors and the blockade of Y2 receptors in regions of the amygdala promoted consistent reductions in ethanol intake by rats [100, 101]. Similar neurobiological responses have been observed in studies with nicotine, psychostimulants and opioids [85]. In humans, polymorphisms in the promoter region of the *NPY* gene have been correlated with alcohol dependence [102], anxiety disorders [103] and resilience to post-traumatic stress disorder [104].

For obvious reasons, the *Npy* gene has been listed as a candidate in the first publication reporting the QTL *Anxrr16* (at that time named *Ofil1* [10]. This gene was already known not to map close to the QTL peak, but rather in its vicinities, about 40 cM away from the peak. After the development of the SLA16 strains, it was possible to determine that *Npy* was located within the transition region mentioned above that bears a mixture of LEW and SHR genetic backgrounds. Therefore, despite the strong arguments pointing to the *NPY* gene as a functional candidate to explain part of the genetic variability observed in anxiety-related behaviors in humans and in animal models, its map location does not allow us to consider it as a strong positional candidate for *Anxrr16*. In the present article, this gene provides an illustration of how neurobiological function and genome position (which sometimes counterbalance each other) can both guide us in the analysis of how strong the evidence on any candidate gene must be to justify it to be kept in a priority list of candidates.

### 8.2 Crhr2

The *Crhr2* gene encodes for the corticotropin-releasing hormone (CRH) receptor 2 (CRHR2), a G-protein-coupled receptor present in the plasma membrane, which is activated by the CRH and urocortin (UCN) and is expressed in the central and peripheral nervous systems [105-108]. Although its function is not well understood, CRHR2 is believed to predominantly mediate a central anxiolytic response, opposing the general anxiogenic effect of CRHR1, which is also activated by CRH [107, 109]. For example, CRHR2 knockout mice showed increased sensitivity to stress, anxious-like behavior [110] and corticosterone release, as well as an impaired recovery from the EPM and OF tests [111].

Yong and colleagues [112], comparing inbred alcohol-preferring (P) and non-preferring (NP) rats, found that P rats had: i) higher levels of corticosterone after 30-minute restraint stress; ii) lower levels of CRHR2 mRNA in the CNS; and iii) lower density of CRHR2 receptors in the amygdala. In addition, DNA sequencing identified polymorphisms in the promoter and coding regions of *Crhr2* gene between P and NP rats. These strains have been used to reveal a major QTL for alcohol preference on rat chromosome 4, whose peak is located near the *Crhr2* gene region [33]. Since these rats also exhibit anxiety differences [113, 114] and the QTL for alcohol preference largely overlaps with *Anxrr16*, the *Crhr2* gene can be considered as a promising candidate for our main anxiety-related QTL.

### 8.3 Snca

The *Snca* gene is responsible for encoding α-synuclein (α-syn). This small protein (14 kD) is predominantly expressed in the CNS and acts mainly in presynaptic nerve endings [115]. The functional role of α-syn was observed in the: i) formation of the soluble N-ethylmaleimide-sensitive factor attachment protein receptors (SNARE) complex [116]; ii) association with SynaptoBrevin-2 (VAMP2) during synaptic vesicle recycling [117]; iii) growth and development of encephalic neuron subsets [118]; iv) modulation of DNA repair [119]; and v) attenuation of neurotransmitter release [120]. Overexpression of α-syn decreases dopaminergic neurotransmission, the activity of tyrosine hydroxylase and dopamine reuptake mediated by the dopamine active transporter (DAT) [121]. α-Syn has also been implicated in movement disorders such as Parkinson’s disease, multiple system atrophy, ADHD and neurodegenerative diseases such as dementia with Lewy bodies [79, 121].

Our group has identified a SNP in the *Snca* 3'-UTR region that varied between LEW and SHR rats. This polymorphism altered the α-syn expression in the hippocampus and appeared to modulate emotionality through dopaminergic mechanisms. LEW rats showed higher concentrations of α-syn mRNA and protein and less turnover of dopamine in the hippocampus, in addition to being more fearful than SHR rats in the OF, EPM and BWB tests [122]. Other studies have shown that the absence of the *Snca* gene was correlated with learning and memory impairments [123] and with decreased impulsivity [124]. Therefore, considering all the evidence mentioned above, we see *Snca* as a good candidate gene to explain the behavioral findings related to emotionality/anxiety, learning/memory and locomotion in novel environments in SHR, LEW and SLA16 rats.

### 8.4 Tacr1

Based on the combination of functional evidence and map position, as well as on the candidate-gene information validated by the RGD [12], one could consider that the most promising candidate gene for the *locus Anxrr16* is *Tacr1*, possibly being a key element for explaining the behavioral profile of SLA16 rats. This gene is responsible for the neurokinin-1 receptor (NK1R), whose highest affinity ligand is substance P (SP). SP is an 11-amino-acid neuropeptide belonging to the tachykinin family [125, 126]. The SP/NK1R complex is widely expressed in the nervous system and is involved in intestinal smooth muscle contraction, pain, emesis, neurodegeneration, inflammation, cancer and cardiovascular regulation [127, 128]. Moreover, this molecular complex has been involved in modulating emotionality-related behaviors and complex psychiatric disorders [126, 129]. For example, NK1R knockout mice have shown a remarkable phenotypic similarity with the symptoms of ADHD: the animals exhibited hyperactivity, inattention and impulsivity [130, 131]. It has also been observed that the genetic deletion or the pharmacological blockade of NKR1 decreases anxious- [132] and depressive-like behaviors [133, 134]. On the other hand, the administration of NKR1 agonists has been shown to induce anxiogenic effects [135].

Effects of the SP/NK1R complex on the behavior of rodents (especially when involving the medial amygdala) have been observed in different anxiety/depression models. For example, such effects have been reported in the EPM [129, 136], ultrasonic vocalization test [129], forced swimming test [129, 137], OF [129, 137, 138], immobilization stress [136, 139] and fear-potentiated startle [140].

In our laboratory, when treated with NKP608 [141], a selective NK1 receptor antagonist, anxiolytic-like effects were seen in SHR male rats tested in the OF but not in the EPM. In contrast, partial anxiolytic effects were observed in LEW males tested in the EPM but not in the OF. Interestingly, LEW females did not respond to these treatments. These results indicate sex- and strain-dependent sensitivity of the LEW and SHR strains following pharmacological manipulation of NK1R, the product of *Tacr1*, which makes it a good candidate gene for possibly explaining the differences in emotionality between the SLA16 and SHR strains.

### 8.5 Oxtr

The *Oxtr* gene encodes for the receptor of oxytocin (OXT), which is present in the central and peripheral systems [142]. OXT is a neuropeptide produced by hypothalamic nuclei and released into the circulation from the neurohypophysis [143]. In addition to its well-known function in childbirth and lactation, OXT can modulate a variety of responses to social and emotional challenges at different life stages in humans [144, 145] and rodents [142, 146-148]. For example, there is evidence showing that OXT attenuates sustained contextual fear, anxiety-like behavior and the HPA axis activity during stress [149-151]. On the other hand, OXT can sometimes produce opposite two-way behavioral reactions, including anxiogenic or anxiolytic behaviors and increased or decreased stress response [152].

Studies carried out with humans have provided evidence that *Oxtr* SNPs are associated with individual differences in the development of psychopathologies and emotional behaviors [153-157], including physical and social anxiety [155]. In rats, a recent study has identified an SNP at *Oxtr* in the Long Evans strain with an effect on maternal care [158]. However, additional preclinical studies are needed to investigate the existence of other *Oxtr* SNPs, as well as the effect of *Oxtr* on emotionally-contrasting rat strains. In this sense, we hypothesize that genetic variants of Oxtr could explain, at least in part, the variety of emotional and behavioral responses observed in SLA16 rats.

### 8.6 Il17ra, il17re and il17rc

The genes *il17ra*, *il17re* and *il17rc* encode transmembrane proteins acting as interleukin-17 (IL17) A, E and C receptors (IL17RA, IL17RE and IL17RC), respectively. Among these receptors, IL17RA stands out, as well as other receptors from this family that form heterodimeric complexes with IL17RA, by mediating the signaling pathways triggered by IL17 family cytokines [159, 160]. By interacting with its receptors, IL17 induces the activation of several signaling pathways mediated by the cytosolic protein Act1 and different TRAF proteins [161]. Once activated, these proteins result in the transcription of inflammatory genes associated with NF-κB and MAP kinase pathways [160]. Additionally, their signaling induces the production of other pro-inflammatory cytokines and chemokines, intensifying the pre-established inflammatory process [162]. Interestingly, in addition to its regulatory role in innate immunity, IL17 has also been associated with reduced blood-brain barrier integrity and activation of microglial cells. This process may result in an increased inflammation in the CNS, implicating the development of depressive disorders and neurodegenerative pathologies [163, 164]. Last but not least, a study by Lima and colleagues [165] has indicated that gamma delta (γδ) meningeal T cells are associated with IL17 production and the development of anxiety-like behavior in mice. As discussed in the next session of the present article, the interactions between emotional traits and various mechanisms of immunological and inflammatory responses open new avenues for the molecular investigation of emotionality-related psychiatric diseases.

### 8.7 Grin2b

Glutamate is largely responsible for excitatory neurotransmission in the mammalian CNS, whose NMDA-like receptors are voltage-dependent and essential for the control of the conductance of Na^+^, K^+^ and Ca^2+^ across the neuronal membrane [166-168]. NMDA receptors are normally organized in tetramers with two NR1 and two NR2 subtypes [167]. The NR2 subtype can have different subunit forms: NR2A, NR2B, NR2C and NR2D. *Grin2b* encodes for the NR2B/GluN2B subunit, which confers functional heterogeneity to NMDA receptors [168], promotes long-term potentiation (LTP), long-term depression (LTD) and neuronal plasticity [169]. Clinical and preclinical research has investigated the relationship between *Grin2b* and several pathologies [170-173], especially ADHD [174-176]. In addition to cognitive processes such as attention, learning and memory [175-178], *Grin2b* and its product have also been shown to affect emotionality/anxiety. For example, both young and adult mice with decreased NR2B protein levels showed anxiolytic-like behavior. Moreover, when treated early with an NMDA receptor agonist, young mice, but not adults, displayed anxiolytic-like behavior [179].

Interestingly, the most widely characterized animal model of ADHD is the SHR strain, which is derived from WKY [180]. Jensen *et al*. [176] have observed that LTP in SHR rats at postnatal day 28 was significantly reduced by the NR2B specific blocker (CP-101, 606), whereas it had no effect in controls. In other strains, using a chronic prenatal stress model, hippocampal expression of *Grin2b* was increased in LEW and Sprague-Dawley, but not in Fischer rats [181].

In preliminary studies from our group, LEW and SHR strains were compared for NR2B gene expression in the hippocampus and hypothalamus [182]. No significant differences have been observed, suggesting that NR2B subunit expression does not differ between these strains and in these brain areas. However, we maintain *Grin2B* as an interesting candidate gene as there is still a need for the investigation of: i) other brain areas; ii) the presence of polymorphisms within the gene; iii) NR2B receptor binding affinity; and iv) LTP and LTD of NMDA receptors. Future experiments evaluating the sensitivity to compounds acting on the NMDA receptor may also help to determine the function of *Grin2b* on the emotionality/anxiety and hyperactivity differences among LEW, SHR, and SLA16 rats.

## 9 IMMUNE SYSTEM: A NEW WINDOW TO THE IDENTIFICATION OF EMOTIONALITY-RELATED QTG?

Nowadays, it is known that immune responses can disrupt emotional well-being and trigger behavioral changes related to persistent psychological stress [183]. This process has been associated with constituents of neuroinflammatory and neuroendocrine character, since genetic variation can modulate the activation of multiple immune cascades involving the HPA axis, resulting in the manifestation of various psychiatric disorders, such as depression- and anxiety-related ones [183-186].

Of particular interest for our research group, the immune system is fundamental for several functions that maintain homeostasis by blocking pathogen invasion, limiting the growth of cancers and rejecting foreign tissues [187]. Moreover, some evidence points out that the immune system plays a role in regulating different CNS functions, even in the absence of acute stimuli [188]. For example, the pro-inflammatory cytokines are known to influence brain functions related to cognition, learning, memory and synaptic plasticity, as well as interacting with neural structures affecting mood, anxiety, motivation and psychomotor activity [189-193]. Therefore, interactions between the immune system and the brain attract considerable attention regarding their role in the development of neuropsychiatric disorders.

Research in the field of “psychoneuroimmunology” shows that several immunological elements are present in CNS diseases, whether of inflammatory origin or not. The presence of cellular infiltrates (T lymphocytes), expression of the major histocompatibility complex (MHC) class II and the production of pro-inflammatory cytokines have already been observed in the context of CNS-related pathologies. Moreover, some cytokines can induce the synthesis of different enzymes, such as Indoleamine 2, 3-Dioxygenase (IDO) and GTP-cycle hydrolase 1 (GTP-CH1) that influence the biosynthesis of neurotransmitters associated with depression and anxiety, including serotonin, norepinephrine, dopamine and glutamate [188, 194-196]. In addition, cytokine activation may also play an important role in the molecular and behavioral responses to antidepressants [197]. Interestingly, some antidepressants have been shown to produce neuroprotective effects through anti-inflammatory mechanisms [198]. Consequently, the regulation of inflammatory responses may involve various and complex neural signaling pathways occurring at genetic, transcriptional, translational and protein-interaction levels.

Studies seeking to create genetic maps of neuroinflammatory profiles in rats have revealed some important *loci*. The QTL *VRA2* (*Neudeg2*) and *VRA3* (*Neuinf1*), both on chromosome 5, seem to be critical in regulating T-cell migration into the CNS. This cellular recruitment can be influenced by several mechanisms, such as peripheral preactivation of T cells enabling their passage across the blood-brain barrier [199, 200] or through the expression of adhesion molecules [201], the levels of cytokines and their respective receptors [202]. These processes are mainly related to the development of primary CNS inflammatory diseases [203].

A *locus* on rat chromosome 10 (*Neuinf2* or *VRA4*) has been involved in the expression of MHC class II [203]. Although little present in the CNS under normal conditions, studies indicate that, in some neurological diseases, there is an increase in the expression of MHC class II complex in the CNS, mainly when associated with the activation of microglia cells [195]. Besides promoting neuronal synapse, these cells can act as an antigen-presenting cell (APC), helping activate T lymphocytes and the propagation of inflammatory reactions [204]. Interestingly, depression, anxiety-related disorders and stress have already been associated with the activation and abnormal increase of microglial cells in the CNS [106, 205]. Additionally, Diez and colleagues [206] have demonstrated that transcription of MHC Class II proteins in rats is strongly regulated by two regions of chromosomes 1 (*Neuinf4*) and 7 (*Neuinf5*) and mildly regulated by three other regions of chromosome 1 (*Neuinf8*), 7 (*Neuinf6*) and 10 (*Neuinf7*). These findings reinforce the high complexity involved in the genetic mechanisms underlying the immune response variability in the CNS.

Of special interest for our laboratory, another study has searched for candidate genes within the QTLs *Eae24-Eae27,* all of which are located on rat chromosome 4, with two (*Eae26* and *Eae27*) overlapping with the QTL *Anxrr16*. These *loci* are known to regulate experimental autoimmune encephalomyelitis, an animal model of neuroinflammatory diseases. The *Eae25 locus* was found to contain genes known to influence both B cell function and T cell activation. Furthermore, modulation of the levels of anti-myelin glycoprotein antibodies of oligodendrocytes (Anti-MOG) and immunoglobulin G1 (IgG1) were associated with the *Eae24* region, while IgG2B antibodies related to both *Eae24* and *Eae26* regions [207]. In rats, IgG1 levels reflect the T2-type immune response, which is categorized as an adaptive immune response of differentiated T helper cells, eosinophil recruitment and antibody production through the secretion of a repertoire of pro-inflammatory cytokines, which include IL4, IL5 and IL13 [208]. Meanwhile, IgG2B levels reflect the T1-type immune response resulting in the secretion of IL2 and IFN-γ [209]. The balance between T1 and T2 system cytokines has a role in modulating cellular responses in the brain during psychological stress processes and depression [197].

In conclusion, some studies have demonstrated the existence of a substantial genetic basis underlying the vulnerability of neurons to immune responses occurring in the CNS. Despite such influences, few studies have sought to map genetic regions responsible for the interaction between immune responses and emotionality. This relatively unexplored territory opens new perspectives concerning the potential identification of a new category of QTLs regulating phenotypes that are relevant to the study of emotionality-related disorders.

## 10 THE CHALLENGES OF QTL MAPPING IN HUMANS

In the past few years, substantial progress has been made in identifying the genetic basis of many hereditary human diseases [210-212]. However, the identification of genetic factors underlying complex pathologies - such as mental disorders - has been much more complicated [213]. There are numerous reports of genes or loci that may underlie emotional disorders in humans, but few of these findings have been replicated.

Approaches focusing on the etiology of mental disorders such as anxiety, depression and mood disorders may advance our understanding of the biological underpinnings of the most common human psychiatric illnesses [214]. Attempts to discover the genetic basis of emotionality-related phenotypes in humans, such as neuroticism (a personality trait characterized by experiencing negative emotions such as anxiety and fear) have revealed extreme complexity. For example, every single item that composes, together with other items, a specific trait of interest, can reveal a unique genetic basis, which makes the study of composite psychological traits very difficult [215].

Since the 1990s and 2000s, several studies have used the assessment of neuroticism through questionnaires, which made it possible to obtain larger sample sizes [216-218]. Continuous self-reporting personality and mood scales in the genetic analysis of affective psychopathology is complementary to clinical diagnoses and may provide greater statistical power [219]. In this period, the methods of linkage analysis between concordant and discordant pairs of siblings were examined for sharing alleles at multiple genome sites defined by genetic markers. The more often affected siblings share the same allele at a specific site, the more likely the site is close to the disease-causing gene [213]. Moreover, linkage analysis of concordant high, concordant low, or discordant sibling pairs selected from a sample maximizes the chance of detecting genetic effects [220].

Linkage analysis on neuroticism, involving highly concordant and discordant sibling pairs, indicated the existence of QTL on chromosomes 1, 3, 4, 6-8 and 11-13 [219, 221, 222]. Some of these analyses identified regions that were potentially sex-specific. In agreement with these results, it is estimated that about 45% of the genetic liability to major depressive disorder (MDD) is not shared between sexes in humans [223, 224]. The linkage analysis method, successfully used to find important genes, has limited power to detect genes of modest effect. Thus, different approaches have been developed for large-scale testing by association analysis, mainly for the genetic study of complex diseases [213].

Over the past 15 years, with the introduction of GWAS combined with reference studies on human genetic diversity and new technologies for large-scale DNA analyses, understanding the genetics of complex human disorders has shown significant progress [225, 226]. Recent studies have sought, for example, to obtain low sequence coverage of many individuals in order to scan the genome for signs of genetic association [224, 227]. Moreover, the recruitment of many individuals with relatively homogeneous cases of serious illnesses has allowed the successful discovery of genetic risk *loci*. A recent mega GWAS has identified 12 loci significantly associated with ADHD [228], some of located at or near genes relevant to the neurodevelopmental processes of ADHD, including *FOXP2*, *SORCS3*, and *DUSP6*. According to Demontis *et al*. [228], the significant genetic correlation between ADHD, MDD and other depressive symptoms suggests a genetic overlap of different psychiatric disorders [229].

## 11 FILLING THE TRANSLATIONAL GAP BETWEEN RATS AND HUMANS

Due to the difficulties of translating phenotypes from animals to humans, the co-localization of behavioral QTL across species is hard to accomplish. However, it remains plausible to assume that a number of behavioral mechanisms, which carry a significant survival advantage, have been conserved throughout evolution. In keeping with such an assumption, highly conserved phenotypes are expected to share many, if not all, underlying genes. Numerous species possess an evolutionarily conserved structure of fear-modulating circuits. Therefore, insights into fear-related QTL location and function can be provided by investigations of QTL concordance across species, with the subsequent assessment of the relevance of the corresponding candidate genes to human pathologies [230]. Thus, the potential congruence of human and animal studies may provide a way to identify new genes that contribute to the susceptibility to various emotional disorders [221]. Examples of congruence have been provided by high-resolution mapping in the mouse that detected several *loci* influencing emotionality on chromosome 1, which were syntenic with human chromosome 1q [231, 232].

A recent study, demonstrated congruence between species using the Collaborative Cross (CC), a mouse model representing a genetically heterogeneous population with evenly distributed allelic variation and human-like allele frequency distribution [233]. This model investigated the complex interaction between host genetics, gut microbiota and anxiety. When a GWAS was carried out to investigate the contribution of genetic variation to anxiety-like behaviors, 264 SNPs and 141 named genes were significantly associated with anxiety. 62 genes have been associated with anxiety, behavioral changes and neurodevelopment. In addition, the 141 identified mouse genes have been compared to a compiled list of human genes associated with seven psychiatric conditions. Significant overlap was found with 25 of the 141 anxiety-related mouse genes associated with one or more psychiatric conditions such as ADHD, depression and neuroticism [233].

In spite of the intense and long-lasting efforts to unravel the molecular pathways that lead to human psychopathologies, the limited preclinical advances seen in the past decades (which were based mostly on the use of rodent models) suggests that translational approaches must move beyond mammalian species. The predominant popularity of fruit flies and mice among geneticists of the 20^th^ century has probably slowed down the development of alternative animal models such as fish, which started to gain visibility mostly in the current century, thanks to the increasing fame of zebrafish (*Danio rerio*).

According to Xu and Guo [234], several attributes of zebrafish suggest that they can be a powerful model for complementing GWAS in humans and rodents. The zebrafish genome is fully sequenced and it shows about 70% of its genes having at least one human orthologue [235]. In addition, more than 80% of the genes related to human diseases have at least one zebrafish orthologue. Comparative genomic analyses between zebrafish and humans have revealed double-conserved synteny (DCS) blocks that are represented in each human chromosome [235]. Taken together, the available biological evidence and recent technological advancements point to the experimental testing of GWAS strategies, with a subsequent translation of zebrafish findings back to humans, as a highly promising tool [234].

The comparative approach of QTL concordance between species increases translational relevance in genetic studies. In this regard, the zebrafish is becoming increasingly popular in behavioral neuroscience and genetics, as this species offers a cheap and simple alternative to classical laboratory rodent models in the quest to understand the mechanisms of human disorders.

Paradigms capable of high throughput and comprehensive characterization, in parallel with advances in genetic and genomic approaches, have advanced our knowledge of the genetic mechanisms underlying the behavioral repertoire exhibited by zebrafish [234]. For example, the zebrafish has been proposed as an excellent animal model for the analysis of anxiety-like behaviors. Several methods have been developed to quantify responses related to anxiety and fear in this species. Numerous results have demonstrated that pharmacologically validated anxious-like responses in mammals work in an expected way in zebrafish, implying evolutionary conservation of processes and, therefore, translational relevance of this species [236].

The first study to use zebrafish in QTL analysis evaluated the behavioral differences between a wild fish strain from Bangladesh and the laboratory strain AB [237]. In total, 184 F2 fish were tested for their shoal tendency and willingness to approach an unknown object (“boldness”). QTL for boldness was found on chromosomes 9 and 16, in addition to another genomic region that influences anti-predator behavior on chromosome 21 [237]. The genetic manipulation of anxiety-related phenotypes in a large, multidisciplinary study exemplifies how multiple species analyses (in this case, zebrafish, mice, and humans) can lead to highly relevant discoveries [236]. A study by Choi *et al*. [238] has revealed a new gene family (*samdori* or *sam*) that encodes chemokine-like factors. Knockout zebrafish carrying a silenced allele of *sam2* (a member of this family) exhibited elevated anxiety responses in the new tank, light-dark and shoaling assays. Such results were then replicated in the *sam2* knockout mouse. Due to the high conservation of nucleotide sequences between the zebrafish and human genes, the authors were able to identify a human homolog of the *sam2* gene and confirm that its bearing region was associated with intellectual disability and autism spectrum disorder in a chromosome deletion syndrome.

## CONCLUSION

Based on the main limiting factors discussed in the previous sections, we can summarize as follows the main “traps” to be avoided in psychopharmacological gene-hunting strategies. 1) Do not expect short-term projects to provide conclusive links between a phenotype of interest and individual genes; 2) Be aware that composite traits (like neuroticism or anxiety) comprise numerous smaller components that may relate to distinct genetic systems. In other words, your QTL is probably small and possibly test-specific; 3) Do not underestimate gene-sex interactions (thus always include both males and females); 4) Major causes of variation may not be observable in your specific set of strains; 5) Your sample is never too large; 6) Do not place too much confidence in a study that has not been replicated; and 7) Remember that the polymorphism you are searching for may be far away from any transcribed region.

As far as the location, identification and functional characterization of genes related to emotional responses are concerned, the past is complex but filled with beautiful examples and promises. The present makes us less naïve but even more hopeful, since we can now understand that collaboration and integration (of areas, methods and species) is not only advisable, but absolutely indispensable. Hurry is not our friend in this field, but history and technology are.

The future is promising and certainly involves the thorough search and identification of Quantitative Trait Nucleotides to be associated with phenotypes that are relevant to psychiatry [239].

Modernly, the effect of genes can be confirmed by creating CRISPR/Cas9 knockout lines. The main idea is to compare those lines with their wild-type genotypes, as CRISPR allows the replacement of specific single nucleotides and the addition or deletion of specific sequences [240, 241]. Also, applying a genome editing system could create novel alleles with larger phenotypic effects at otherwise minor QTLs, which can be an effective way to causally validate genes and gene networks [242]. Yet, we must not neglect or underestimate the foremost importance of phenotype measurement, analysis and interpretation. The most powerful molecular tools will tell us nothing if we don’t master, standardize and refine our behavioral analysis methods aiming at clinical translation. In other words, we still need, more than ever, rigorous behavioral neuroscience.

Finally, the slow but continuous progress achieved so far in the identification of genes related to emotionality in animal models will, sooner or later, open new perspectives in the psychiatric treatment of human beings.

## Figures and Tables

**Table 1:** Studies using different strains of rats and their intercrosses in Quantitative Trait Loci (QTL)/Quantitative Trait Gene (QTG) analyses related to emotionality, hyperactivity or stress.

**Phenotype**	**Tests**	**Design**	**Strains**	**QTL/QTG Name**	**Chr**	**References**
Stress	Immobilization stress	Congenic	RIS, BN.L.x	*Sradr5*	7	[50]
		Congenic F1 F2	BN WKHA Wistar	*Stresp14*	7	[39]
				*Stresp16*	3	
		F2	hHTg, BN	*Stresp6, Stresp7*	10	[51]
		Congenic	WKYpch10, WKYpch1.1	*Cstrr1*	1	[52]
	OF, FST, DB	F2	WKY F344	*Sradr1, Bp377, Bp307*	1	[53]
				*Srcrt1, Sradr2*	2	
				*Srcrt2, Srcrtb1*	3	
				*Sradr3, Srcrt3*	4	
				*Srcrtb2*	5	
				*Srcrt4*	6	
				*Sradr5*	7	
				*Srcrt5*	15	
				*Sradr6*	18	
	DB	Congenic F2	WKY F344	*Stresp1, Stresp2, Stresp3*	X	[54]
				*Stresp8*	7	[55]
				*Stresp10*	6	
				*Stresp11*	8	
				*Stresp12*	13	
	Contextual fear conditioning, OF, DB	Congenic	F344.WKY-Stresp10 F344 WKY	*Stresp10*	6	[56]
				*Gpatch11, Cdkl4, Drc1*	QTG	
		Congenic	ISIAH/Icgn WAG/Gsto-Icgm	*Bp46*	18	[57]
	ADX	F2	BN, F344	*Gmadr1*	19	[58]
Anxiety Emotionality Hyperactivity	OF, EPM, AC	Congenic F2 F3-F5	WKY WKHA LEW SHR/NIco BN.Lx/Cub SHR/Ola HXB/BXH	*Anxrr16*	4	[10]
				*Anxrr17*	7	
				*Activ1*	8	[31 , 59]
				*Anxrr17*	7	[60]
				*Anxrr3, Anxrr7*	2	[61]
				*Anxrr6, Anxrr8*	7	
				*Anxrr4*	5	
				*Anxrr5*	6	
				*Anxrr9*	8	
				*Anxrr10*	9	
	OF, EPM, Spontaneous activity, ASR, Classical Fear Conditioning, Two-way avoidance	F2	RHA/Verh RLA/Verh	*Anxrr21*	3	[35]
				*Anxrr22*	5	
				*Anxrr23*	6	
	OF, EPM, AC, Two-choice saccharine, Two-choice quinine	Congenic	LEW/CRLIFO SHR/CRL	*Anxrr16*	4	[62]
	OF, FST, DB	Parental F1 F2	WKY F344	*Anxrr18*	2	[63]
				*Anxrr19*	10	
				*Anxrr20*	18	
	OF, EPM, BWB, Triple test, T-maze, Home cage, AC, Attentional set-shifting	Congenic	LEW, SHR, SLA16	*Anxrr16*	4	[64]

**Abbreviations**: Chr = Chromosome. Strains: RIS = Recombinant inbred strains; BN = Brown Norway; WKHA = crossing of SHR and WKY; hHTg = Hereditary Hypertriglyceridemic; WKY = Wistar Kyoto; F344 = Fisher; ISIAH = Inherited Stress-Induced Arterial Hypertension; WAG = Wistar Albino Glaxo; LEW = Lewis; SHR = Spontaneously Hypertensive Rats; HXB/BXH = inbred SHR/Ola and BN.Lx/Cub; RHA and RLA = Roman High and Low Avoidance rats; SLA16 = SHR.LEW-Anxrr16. Tests: AC = Activity cage; DB = Defensive Burying test; OF = Open-Field; EPM = Elevated Plus-Maze; BWB = Black and White Box; FST = Forced swimming tests; ADX = Adrenalectomy; ASR = Acoustic Startle Response.

**Table 2:** Nine relevant genes were mapped in the differential genomic region (DGR) of SLA16 *vs.* SHR rats.

**Gene**	**Gene Product**	**Position (Mbp)**	**References**
*Npy*	Pro-neuropeptide Y preprotein	78.9	[82]
*Crhr2*	Corticotropin releasing hormone receptor 2	84.2	[82]
*Snca*	Alpha-synuclein	89.7	[82]
*Tacr1*	Substance-P receptor	114.9	[12]
*Oxtr*	Oxytocin receptor	145.6	[12]
*Il17re*	Interleukin 17 receptor E	146.6	[12]
*Il17rc*	Interleukin 17 receptor C	146.6	[12]
*Il17ra*	Interleukin 17 receptor A	153.7	[12]
*Grin2b*	Glutamate receptor ionotropic N-methyl-D-aspartate Subunit 2B	168.6	[82]

## LIST OF ABBREVIATIONS

ADHD: Attention Deficit Hyperactivity Disorder
APC: Antigen-presenting Cell
BN: Brown Norway
BWB: Black and White Box
CC: Collaborative Cross
CNS: Central Nervous System
DAT: Dopamine Active Transporter
DB: Defensive Burying
EPM: Elevated Plus-maze
FC: Fear Conditioning
GWAS: Genome-wide Association Studies
HRDP: Hybrid Rat Diversity Program
IDO: Indoleamine 2, 3-Dioxygenase
LTD: Long-term Depression
LTP: Long-term Potentiation
MDD: Major Depressive Disorder
OF: Open Field
OR: Object Recognition
QTG: Quantitative Trait Genes
QTL: Quantitative Trait Locus
RGD: Rat Genome Database
SA: Spontaneous Alternation
SHR: Spontaneously Hypertensive Rats
SNP: Single Nucleotide Polymorphisms
WKY: Wistar Kyoto
